# Morphological encoding beyond slots and fillers: An ERP study of comparative formation in English

**DOI:** 10.1371/journal.pone.0199897

**Published:** 2018-07-25

**Authors:** Harald Clahsen, Silke Paulmann, Mary-Jane Budd, Christopher Barry

**Affiliations:** 1 Potsdam Research Institute for Multilingualism (PRIM), University of Potsdam, Potsdam, Germany; 2 Department of Psychology & Centre for Brain Science, University of Essex, Colchester, United Kingdom; 3 School of Psychology, University of East London, London, United Kingdom; Leiden University, NETHERLANDS

## Abstract

One important organizational property of morphology is competition. Different means of expression are in conflict with each other for encoding the same grammatical function. In the current study, we examined the nature of this control mechanism by testing the formation of comparative adjectives in English during language production. Event-related brain potentials (ERPs) were recorded during cued silent production, the first study of this kind for comparative adjective formation. We specifically examined the ERP correlates of producing synthetic relative to analytic comparatives, e.g. *angrier* vs. *more angry*. A frontal, bilaterally distributed, enhanced negative-going waveform for analytic comparatives (vis-a-vis synthetic ones) emerged approximately 300ms after the (silent) production cue. We argue that this ERP effect reflects a control mechanism that constrains grammatically-based computational processes (viz. *more* comparative formation). We also address the possibility that this particular ERP effect may belong to a family of previously observed negativities reflecting cognitive control monitoring, rather than morphological encoding processes per se.

## Introduction

This study presents results from event-related brain potentials (ERPs) elicited by ‘morphological encoding’, that is the processing of morphologically complex words during language production. One prominent account of morphological encoding is the slot-and fillers model [1], which is essentially an implementation of affixation, with an additional adjustment for non-affixal (e.g., suppletive) morphology. For instance, both regular and irregular past-tense forms (e.g., *walked*/*fell*) are thought to require a two-slot inflectional frame, but while for regular past-tense forms two phonological codes will be retrieved, only one single phonological code will be retrieved for irregular forms. However, the production of morphologically complex words involves more than filling slots for stems and affixes. One core property of morphology is competition [2], with different morphological exponents competing for expression. A well-known case of morpho-lexical competition is that general processes need to be constrained to avoid speech production errors (e.g., *gloriousness or *fighted instead of *glory* and *fought*). As pointed out by Aronoff (2016) [2], morphological systems resolve competition in different ways. One of the two competitors might emerge as a default, with other ones left as lexical exceptions, as for example, in the case of regular versus irregular past-tense inflection in English, where the regular–*ed* functions as the default and irregular forms as exceptions [3]. Division of labour in morphology is also quite common, for example, *un*- vs. *in*- prefixation in English, where *in*- and its variants are restricted to words of Latin or Greek origin and *un*- applies to Germanic words.

Competition between different resources is common to language-production processes at all levels of linguistic encoding, not only for morphological processes. Much previous research has been devoted to competition during single spoken word production; see [4] for a review. Competition in word production is thought to arise from the speaker having to select the target word from a set of co-activated words related to the target. The most commonly used experimental paradigm in this line of research is the picture-word interference task in which participants being presented with pictures of objects and a superimposed written distractor word have to name the picture and ignore the distractor; see [5] for recent use with EEG. This paradigm has yielded a number of robust and replicable effects, which have informed models of spoken word production. One common finding are interference effects, i.e. enhanced picture-naming latencies (relative to an unrelated control condition) when the target and the distractor words belong to the same semantic category (e.g. *dog/cat*) and facilitation when the two words are semantically associated (e.g. *dog/fur*). Evidence from ERPs confirms this contrast obtained from behavioral studies in that semantically associated distractors (unlike distractors of the same semantic category) produced a reduced negativity (relative to unrelated words) on picture naming indicating facilitated object identification ([6]).

Over recent years, insights into the brain networks involved in the control of competition during language production, that is the ability to regulate this process, have come from a number of studies. A focus of this research has been language control in bilingual populations, typically examining this phenomenon using picture-naming tasks. These studies have led to the identification of a widely distributed neural network orchestrating the activation of one of a bilingual person’s languages whilst inhibiting the other [7–8]. This network engages the pre-supplementary motor area in the dorsal anterior cingulate cortex, the left prefrontal cortex, the left caudate and the inferior parietal lobules bilaterally together with control input from the right prefrontal cortex, the thalamus and the putamen of the basal ganglia and the cerebellum (see [9], for a recent review).

Electrophysiological studies have provided crucial information about the time-course of bilingual language control in picture naming and other tasks (see [10], for a review). Christoffels, Firk and Schiller (2007) [11], for example, identified an enhanced fronto-central negativitiy (‘N450’) between 350ms and 550ms in response to bilingual language control (see also [12–13]). A comparable negative modulation following the same time-course and scalp distribution was also reported when inducing lexical competition in monolinguals [14]. In that study, semantically related distractors led to higher N450 amplitudes than semantically unrelated distractors in a phoneme monitoring task, possibly reflecting increased competition caused by activating multiple concepts in semantically related trials. In fact, amplitude modulations of this fronto-central negativity have been argued to indicate competition processes between different linguistic resources during language production [15]. Another ERP component that has been linked to competition is the ‘N200’, a frontally distributed negativity that is typically elicited in cases of competition in go/no go tasks, which require responses for one class of stimuli (‘go’) but not for another class of stimuli (‘no-go’). In language-related tasks, this negativity is observed relatively late, between 300ms and 700ms [16–19]. Rodriguez-Fornells et al. (2005) [17], for example, studied phonological competition on bilingual picture naming in one language from a bilingual’s other language in a go/no-go task. An enhanced negativity with a frontal maximum was found between 300 and 600ms in cases of competing phonological codes in the two languages as compared to congruent trials. Although the functional significance of these fronto-central negativities is not yet entirely clear, increases in amplitude of these negativities seem to be related to the control and monitoring of competing resources (see [20], for further discussion).

Competition is also a core element of morphological encoding, which, for the current study, was examined by recording ERPs during the production of complex word forms. ERP studies of morphological processing in adults have previously relied on priming and violation studies, which require secondary (typically metalinguistic) skills, such as acceptability judgments or lexical (word/non-word) decisions. While production tasks have the advantage of tapping into a primary linguistic skill, muscle activation involved in articulation during overt production may distort the EEG signal. One way of largely avoiding such artefacts is the technique we employed for the present study–following Budd et al. (2013) [21]–in which participants are prompted to first *silently* produce the target word form and only thereafter to overtly produce it. By time-locking the EEG to the silent production stage, this design provides insight into planning the production of morphologically complex words prior to overt articulation. Four previous ERP studies used a silent production-plus-delayed-vocalization-task to examine different kinds of morphological phenomena: regular and irregular past-tense forms in English [21–22], regular and irregular plural forms in English compounds [23] (Budd et al., 2015), and regular and irregular past-participle forms in German [24]. These studies yielded consistent results: an enhanced negativity most often particularly pronounced at right frontal electrode-sites elicited during the (silent) production of regular (relative to irregular) word forms in a similar time window (i.e., between 300 to 450ms). This brain response has been interpreted as signaling morphological encoding, specifically the composition process involved in forming an inflected word form through affixation, for example [walk + -ed], which is required for regularly inflected word forms but not for irregular ones (see [21]). Note, however, that this interpretation crucially relies on ‘affix (de)composition’, a concept that is controversial in psycholinguistic research on morphological processing (see [25] for a recent review). Consider therefore an alternative functional interpretation for this frontal negativity that is less dependent on the notion of affix (de)composition. The core idea is that the negativity may signal competition between different morphological forms for a given function during production. In the particular case examined by Budd et al. [21] a highly productive regular morphological process (viz–*ed* past tense formation) needs to be constrained so that it does not over-generate and does not output incorrect forms such as *holded* (instead of *held*). Applied to production, we can think of such a constraint as an output filter. Assume a dual-morphology model for producing morphologically complex words (e.g., [26]), with a look-up component for retrieving (irregular) forms stored in lexical memory and a rule-based component for forming productive (regular) inflected word forms. The output of the lexical look-up component is unconstrained, that is, once a specific entry (e.g., *held* as the past-tense form of *hold*) is found, this entry is submitted to the speech output system and articulated. By contrast, the rule-based mechanism needs to be constrained to ensure that there is no competitor from the lexical look-up system that blocks the candidate rule-based form. It is possible that the morphological encoding negativity reflects this additional control process involved in producing correct regularly inflected word forms.

The specific linguistic phenomenon we examined in the present study is comparative adjective formation in English, which provides an opportunity to decide between these two functional interpretations of the morphological encoding negativity. The production of comparative adjectives involves competition between synthetic and analytic means of expression, as gradable adjectives can form comparatives both synthetically by–*er* suffixation (e.g., *big—bigger*), and/or analytically by a periphrastic form with *more* (e.g., *important—more important*). In addition, a small number of highly frequent adjectives have suppletive comparative forms (e.g., *good–better*, *bad–worse*). There is no semantic or interpretational difference between these means of expression [27]. Descriptive grammars of English [28–29] noted a division of labor between the analytic and the synthetic comparative forms, with the latter applying to short (monosyllabic) adjectives and the former applying to long (multisyllabic) ones. Hilpert (2008:399) [30] stated that monosyllabic comparatives have a ‘nearly uniform tendency to form only one variant’, namely the synthetic form. This contrast, however, only represents a rough trend, as there are many exceptions, such as disyllabic adjectives ending in–*y* (e.g. *happy*) or–*ow* (e.g. *shallow*) that readily allow -*er* comparatives. Furthermore, some monosyllabic adjectives commonly appear with *more* comparatives (e.g. *apt*, *lax*, *chic*). In a recent acceptability rating study, LaFave (2015) [31] found that many monosyllabic roots (typically of Germanic origin) do indeed prefer the synthetic form (*defter*) over the analytic form (*more deft*). On the other hand, this was not the case for many other monosyllabic adjectives—typically of non-Germanic origin and low in frequency–for which the analytic comparative form was rated either better or equally well as the synthetic form, a finding that casts doubt on the conventional grammatical wisdom that the two comparative forms are in complementary distribution with the synthetic form reserved for short and the analytic one for long adjectives; see Aronoff and Lindsay (2015: 6) [32] for further discussion.

As an alternative, it has thus been proposed [33–34] that the synthetic comparative forms are a closed class of items–likely to be stored in lexical memory—whereas analytic comparatives apply by default unless blocked by a lexically listed comparative form. As Poser (1992: 18) [33] put it: ‘If the lexical form [the synthetic one, in our terms] exists, the category is instantiated and so the periphrastic form [the analytic one in our terms] is *blocked* [our emphasis].’ This contrast may be linguistically represented in terms of a lexical feature [+S(ynthetic) C(omparative)] on those adjectives that take–*er* forms, a feature that is left unspecified on adjectives that take analytic comparatives [35]. From this perspective, English comparative adjective formation qualifies as a case of morphological competition. Furthermore, synthetic versus analytic comparative formation shares with irregular vs. regular inflection the fact that in both cases a lexically specified form competes with a grammatical default process.

With regard to neuro-cognitive research on the processing of morphologically complex adjective forms, there are three previous studies testing adjectives in German. One MEG study compared neuronal responses to existing, synonymous and illegal derived adjective forms ([36]). Two ERP priming studies ([37]; [38]) determined brain responses for inflectionally related word forms as opposed to brain responses for purely lexically-related forms. For the phenomenon the current study examines, however, both experimental psycholinguistic and neuro-cognitive research is rare. To our knowledge, there is only one study that examined the comprehension of synthetic and analytic comparatives ([39], chapter 2) using a self-paced reading experiment. The specific finding from this experiment was that for adjectives that permit both comparative forms, readers tend to prefer the analytic over the synthetic form, a contrast that is consistent with a comparative system in which analytic forms act as defaults. Previous findings on comparative adjective forms in English mainly come from analyses of (mostly written) corpora (see [30], for review)–and from behavioral elicited production tasks (e.g., [34]). One consistent finding from this line of research is that synthetic (-*er*) comparatives are more commonly produced for high frequency monosyllabic adjectives than for low frequency ones, both by adults and children. In line with this finding, speakers were also shown to strongly prefer the synthetic comparative for adjectives that have short lexical decision times, whereas they tended to favor the analytic comparative for adjectives that have longer lexical decision times (RTs larger than 600 ms; see [40]). These frequency and lexical-decision time effects are consistent with the view that synthetic comparative forms are lexically stored ([41]). Furthermore, it was found that both analytic and synthetic comparative forms are subject to children’s overgeneralization errors ([39]; [42]), e.g. *dangerouser and *more fast. Another finding from the elicited production studies with children was that children of all age groups were more accurate in their production of adjectives requiring–*er* forms than those requiring *more* comparatives, probably because children are more familiar with the kinds of short high-frequency adjectives that typically take -–*er* comparatives than with adjectives that require analytic comparatives (see e.g., [34], [43]).

Here, we will use the case of comparative adjective formation to further elucidate the nature of the morphological encoding negativity reported in previous research. If the process of combining a stem with an affix elicits this brain response, as originally proposed by Budd et al. (2013) [21], we should find an enhanced negativity for producing synthetic (–*er*) comparatives, relative to analytic (*more*) forms which do not involve affixation. Alternatively, the morphological encoding negativity may signal competition, ‘rival realizations of the same morphosyntactic meaning’ as Aronoff (2013: 1) [2] put it. In this case, we should find the opposite pattern, an enhanced negativity for producing *more* comparatives relative to–*er* forms, similarly to what was found for regular past-tense formation (relative to irregulars). The current study attempts to adjudicate between these alternatives.

## Methods

### Participants

Twenty right-handed adult native speakers of British English (9 men, mean age 26.7 years, range 19–45 years), all with normal or corrected-to-normal vision, were tested for this study. One participant had to be excluded due to recording problems. All participants gave informed written consent before completing the study, which was ethically approved by the University of Essex Science and Health Faculty Ethics Board.

### Materials

In addition to the two critical conditions of analytic and synthetic comparatives, two additional control conditions were included. This is because–*er* and *more* comparatives do not only differ with respect to their morphological exponents but also in a number of non-morphological ways. A comparison of the brain responses for the production of *happier* and *more neutral*, for example, also includes different lemmas and expressions that differ in word length. To control for these differences, we added two conditions for each of the adjectives used for the critical (comparatives) conditions. One control condition required the production of–*ly* adverb forms (e.g., *happily/neutrally*) and the other the production of negated adjectives (e.g., *not happy/not neutral*). In this way, the potential contribution of the lexical and length differences between synthetic and analytic comparative forms can be properly assessed.

Each participant was presented with 90 different adjectives, 45 that typically form synthetic (-*er*) comparatives and 45 that more commonly have analytic (*more*) comparative forms, henceforth ‘ER-adjectives’ and ‘MORE-adjectives’ respectively; see S1 and S2 Files for a complete list of adjective stimuli. The distinction between ‘typical’ ER-adjectives vs. ‘typical’ MORE-adjectives was based on informal judgements from 10 adult native speakers of English (who did not part in the main experiment). The 90 selected adjectives were presented three times, once to elicit production of a comparative form, once for production of a (-*ly*) adverb form, and once for the production of a negated adjective form with *not*. Thus, 270 items were presented in total. The 45 ER-adjectives were matched with the 45 MORE-adjectives on both word frequency and word length as closely as possible. Frequency counts were taken from the CELEX lexical database [44], specifically the Cobuild frequency count of occurrence per million within a 17.9 million spoken word corpus (‘CobMln’; http://celex.mpi.nl/help/ewordforms.html). The ER-adjectives had a mean word frequency of 44 per million (SD: 55) and the MORE-adjectives had a mean word frequency of 43 per million (SD: 60). A paired samples t-test showed no significant difference between the ER- and MORE-adjectives on the log transformed CELEX word frequencies (*t*(44) < 1). With respect to length matching, recall that adjectives with synthetic comparatives tend to be shorter than those that take analytic comparative forms. This was also reflected in the adjectives selected for the present study. We tried to keep the difference in length as minimal as possible, with the ER-adjectives consisting of one and two syllables (M = 1.4; SD = 0.5) and the MORE-adjectives of two syllables except for two three-syllable items (M = 2.0; SD = 0.3), but overall the ER-adjectives were still significantly shorter than the MORE adjectives (*t*(88 = 7.5, p< 0.01). Furthermore the comparative forms to be produced also differed in length as the ER-adjectives require one-word responses and MORE-adjectives two-word responses. As mentioned above, there were two additional conditions (adverbs, negation) to control for these differences.

### Procedure

All participants were tested in a quiet room at the University of Essex, Colchester, UK. The task was introduced to the participants and the experiment started with 18 practice trials (which were not used for analysis). Each trial began with the presentation of a centred fixation-cross for 200ms, followed by the visual presentation of the adjective (e.g., *slow*) in the centre of the screen for 1000ms, in comic sans, 96-point size font in black on a white background. This was followed by a blank screen, which varied in duration (400, 600, or 800ms). The presentation duration of the blank screen was counterbalanced across conditions. Following this, the silent production cue was presented for 2500ms; this was one of three pictures on which participants were trained prior to the experiment, a black square as a cue to produce a comparative form, a red one for a negated adjective with *not*, and a blue one for (-*ly*) adverbs. ERPs were time-locked to the onset of this silent production cue. The silent production cue was followed by a 2000ms long presentation of a loudspeaker picture to cue overt production of the targeted word forms. An inter-stimulus interval (blank screen) was presented for 1500ms. Participants received 18 practice trials at the beginning of the experiment using adjectives that were not included in the main experiment. In the main experiment, trials were pseudo-randomized and distributed over 6 blocks (45 items each). Each block was followed by a short break. Participants were asked to minimize eye and muscle movements during silent production of words. The run-time of the experiment was approximately 25 minutes. An experimental session (including EEG setup) lasted for approximately 75 minutes.

### EEG recording and data analysis

EEGs were recorded using Neuroscan (version 4.5) acquisition software, from 64 electrode sites according to the international 10–20 system using Ag/AgCl sintered electrodes embedded in an elastic cap (Quik-Cap, Neuromedical Supplies). Bipolar horizontal and vertical electro-oculograms (EOGs) were recorded for artefact rejection purposes. Epochs were extracted from 200ms before the onset of the silent production cue up to 1000ms after cue onset. Recordings were referenced online to the left mastoid. Signals were recorded continuously with an on-line band-pass filter between 0.1 and 70 Hz and digitized at 500 Hz. Electrode impedances were kept below 5KΩ. Recordings were re-referenced off-line to the average of the left and right mastoid electrodes, band-pass filtered between 0.1 Hz and 30 Hz, and baseline corrected. For graphical illustration purposes only, grand average ERPs are smoothed with a 7 Hz low-pass filter.

The EEG data was processed with EEGLAB [45]. To remove typical muscle and eye movement artefacts, an independent component analysis (ICA) algorithm (Infomax) was applied to the data. Additionally, trials with artefacts were identified visually and removed. Trials for which a participant’s overt production was inappropriate were also not included in the ERP analysis. These included productions of another inflected form of the target adjective or the target inflected form of a different lexeme. The rates of unexpected responses in participants’ overt spoken productions were low, indicating that they were able to accurately perform the experimental task. Of the whole data set, there were 2.1% non-target responses, which were not further analysed. The average percentages of unexpected responses were 3% for synthetic comparatives, 7% for analytic comparatives, and 1% for both–*ly* adverbs and negation with *not*.

The negativity of interest has been reported in four previous studies [21, 22, 23, 24] which all showed a frontally distributed effect. Visual inspection of the data confirmed a similar distribution in the present study. We thus focused our statistical analysis on frontal and midline electrode sites and included electrodes from left frontal (LF: F1, FC1, F3, FC3, F5, FC5), right frontal (RF: F2, FC2, F4, FC4, F6, FC6), and midline (ML: FZ, FCZ, FPZ) sites. Time windows of interest for mean amplitude quantification of the ERP data were also guided by these four previous studies [21, 22–24], although visual inspection revealed a longer lasting component in the present data set than previously observed. The time-window of interest was therefore extended to cover the full temporal breadth of the negativity; hence, we extracted mean ERP amplitudes between 300-800ms after stimulus onset. ERP mean amplitudes were analysed using repeated-measures ANOVAs, with the factors *Form* (comparative, adverb, negation), adjective *Type* (ER-adjective, MORE-adjective), and *ROI* (LF, RF, ML).

## Results

**Fig 1:**
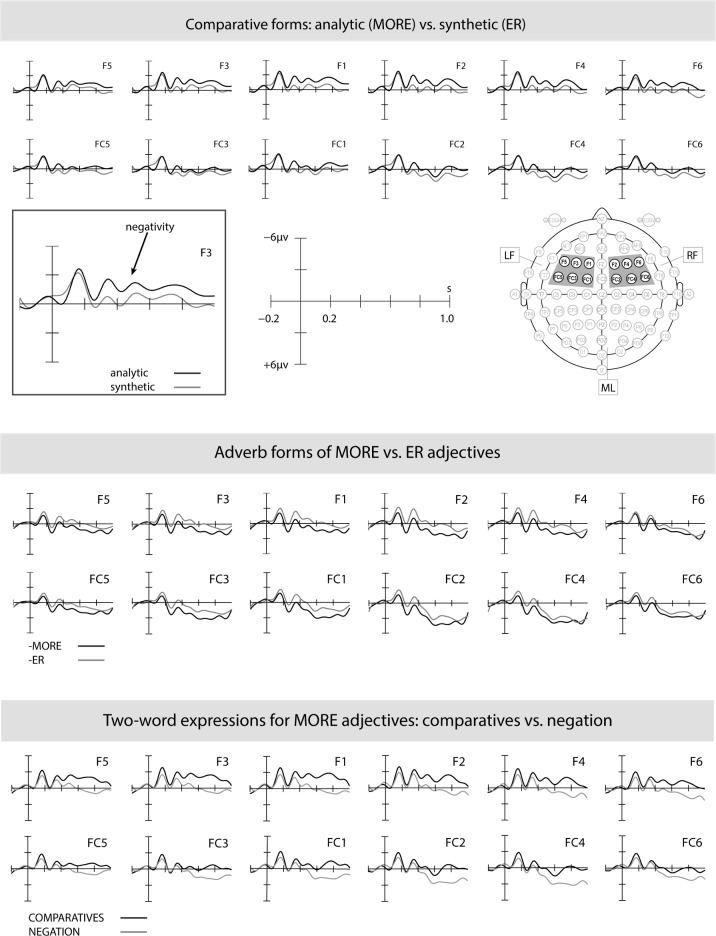
ERP effects for analytic (*more*) vs. synthetic (-*er*) comparatives (top panel), -*ly* adverbs of the different (ER vs. MORE) lemmas (middle panel), and for two-word expressions (MORE+ adjective vs. NEG+adjective) (bottom panel).

For each participant, mean ERP amplitudes were extracted for each electrode site. After data cleaning and after removing incorrect responses, 80.02% of all trials were included in the statistical analysis. Similar numbers of trials were included for the two comparative conditions and the adverb and negation control conditions. The behavioural responses from the delayed overt production task confirmed our expectation that synthetic comparative forms are preferred for ER-adjectives and analytic comparative forms for MORE-adjectives: 97% of the comparative responses to ER-adjectives were with synthetic–*er* forms (816/842), and 92.5% of the comparative responses to MORE-adjectives were analytic *more* forms (781/844). For the analysis of the ERP data of the comparative conditions, we only included trials that yielded the expected responses, i.e. ER-adjectives with synthetic forms, and MORE-adjectives with analytic forms. The grand-average ERP waveforms for comparatives, adverbs and negation can be seen in Fig 1 (top, middle, bottom respectively).

The top panel of Fig 1, which compares the waveforms for the two critical conditions, analytic (*more*) vs. synthetic (-*er*) comparatives, indicates a negative-going waveform elicited when silently producing analytic comparatives relative to synthetic ones between approximately 300ms and 800ms. This waveform is most pronounced at right fronto-central sites. The middle panel of Fig 1 examines whether the contrast seen in the top panel of Fig 1 could be lexical in nature, due to the different lemmas involved. To this end, the middle panel of Fig 1 compares the waveforms for the adverb control condition in which the two types of lemma (ER vs. MORE) had to be produced with the -*ly* adverb suffix. The middle panel of Fig 1indicates that the negativity for analytic comparatives seen in the top panel of Fig 1 is not found for the silent production of–*ly* adverbs of MORE-adjectives, suggesting that the negativity is unlikely to be due to lexical differences between the different lemmas. The bottom panel of Fig 1 examines whether the contrast seen in the top panel of Fig 1 could result from the fact that analytic comparatives require the production of two-word expressions, unlike synthetic comparatives which are single words. To this end, the bottom panel of Fig 1 contrasts the production of analytic comparatives with the production of negated forms with *not*, both of which are two-word expressions and both involve the same lemmas. The bottom panel of Fig 1 displays ERP responses which are similar to the ones seen in the top panel, i.e., a more negative-going waveform for analytic comparatives between approximately 300 and 800ms after the silent production cue. This suggests that the length of response (two word vs. one word) is an unlikely source for the contrast between analytic and synthetic comparatives seen in the top panel of Fig 1.

The statistical analyses confirmed the observations reported above. An ANOVA of the ERP data for the 300 to 800ms time window revealed a significant main effect of *Form* (*F* (2, 36) = 3.96, *p* = .029) but not of ‘Type’ (*p* > .45), and more importantly, a significant interaction between *Form* and *Type* (*F* (2, 36) = 3.58, *p* = .046). Planned follow-up analyses by *Form* confirmed that the negativity for analytic comparatives (relative to synthetic ones) seen in the top panel of Fig 1 is reliable (*F* (1, 18) = 5.55, *p* = .030), whereas there were no significant differences for the ERP waveforms in the middle panel of Fig 1 between *-ly* adverbs for MORE vs. ER-adjectives (*p* > .25). Planned follow-up analyses by *Type* revealed no reliable differences in brain responses for ER-adjectives between the production of comparative and negated forms (*p* > .32). For MORE-adjectives, however, results confirmed the negativity seen in the bottom panel of Fig 1 for the production of analytic comparatives (relative to negation) in the 300 to 800ms time window (*F* (1, 18) = 9.38, *p* = .007).

Analytic (*more*) and synthetic (-*er*) adjectives differ on a number of parameters (with the latter being more frequent and shorter than the former, and typically simplex rather than derived); thus, the ERP responses to the production of these two kinds of adjective forms might also be compared indirectly (with a separate neutral form as a control condition for each of them), rather than directly as done in the analyses above. This can help to further rule out potential length, frequency and complexity effects. To address this, we additionally conducted a 6 (condition) x 3 (ROI) analysis. This revealed a main effect of *condition*, (*F* (5, 90) = 3.15, *p* = .0210), which was followed up with planned post-hoc comparisons. We specifically took the–*ly* adverb condition as a control for–*er* forms and the negation condition as a control for *more* forms, in both cases comparing the same lemmas, e.g. *quietly* vs. *quieter* and *not current* vs. *more current*. As expected from the direct comparisons of–*er* and *more* forms reported above, significant differences between silent production of analytic (*more*) comparative forms vs. negated forms (*F* (1, 18) = 9.38, *p* = .0067) were found, while the planned contrast between synthetic (-*er*) comparative forms and -*ly* adverbs did not reach significance (*F* (1, 18) = 0.09, *p* = .77). This contrast confirms the negativity we obtained in our initial analysis for the silent production of analytic (relative to synthetic) comparative forms.

Summarising, we found an enhanced frontal negativity starting 300ms after silently producing analytic (*more*) comparative forms relative to synthetic (-*er*) ones. Lexical differences between the adjective lemmas and length differences between the two comparative forms could be ruled out as potential sources for this ERP effect.

## Discussion

Language production involves competition between different resources for linguistic expression, and the question of how the brain controls competition during language production has received considerable attention in the psycholinguistic and neurocognitive literature, particularly but not exclusively with respect to how the brain orchestrates inhibition and control of multiple languages in bilinguals. The current study examined competition during the production of morphologically complex expressions (morphological encoding), by testing analytic vs. synthetic comparative adjective formation in English (e.g., *quiet—quieter* vs. *current–more current*). In terms of their surface forms, synthetic comparative forms (e.g., *quieter*) are fully transparent, but they are lexically restricted and represent a largely closed class of items. Analytic comparative formation, on the other hand, has been argued to be a default process which applies unless blocked by a lexical feature [+S(ynthetic) C(omparative)] on a listed set of adjectives that take synthetic forms [33–34]. The results of the current study indicate how the brain responds to this case of morphological competition. We propose that the enhanced negative ERP for the (silent) production of analytic comparative forms reflects the additional constraint that controls the output of a default process (relative to lexicalized comparative forms).

In the following we will discuss the temporal sequencing, the spatial distribution, and the functional significance of the enhanced negativity obtained in the current study in the light of previous neurocognitive studies on morphological encoding.

As regards the *temporal sequencing* of processes involved in language production, brain potential studies of morphologically complex words have led to the identification of morphological encoding as a distinct component within the temporal sequencing of processes involved in language production. Koester and Schiller (2008) [46] examined the production of Dutch compounds in a primed picture-naming task and reported ERP responses to morphological encoding from 350ms onwards after the visual stimulus. This estimated timing of morphological encoding in speaking is consistent with the results of a meta-analysis of 82 picture-naming experiments [47] which identified morphological encoding as taking place between approximately 250-330ms after the visual stimulus, which is after semantic encoding (~250ms) but before phonological encoding (~330-450ms). In accordance with this timeline, Sahin et al. (2009) [48], reported effects of morphological encoding between 320ms and 450ms after cue onset. Furthermore, Lavric, Pizzagalli, Forstmeier and Rippon (2011) [49] also obtained ERP signals of morphological encoding for the generation of English past-tense forms in a similar time window, from approximately 300ms onwards with increased activity in right frontal and temporal brain areas, particularly for generating regular–*ed* (relative to irregular) forms. Finally, four ERP studies using the silent-production-plus-delayed-vocalization task [21, 22–24] reported an enhanced frontal morphological encoding negativity between 300 and 450ms post onset for different kinds of morphological phenomena (past tense, plurals, participles), different languages (English, German), and different populations (native and non-native speakers). Our current ERP findings are thus broadly consistent with the timing of morphological encoding processes reported in previous studies and proposed in models of speech production.

**Fig 2:**
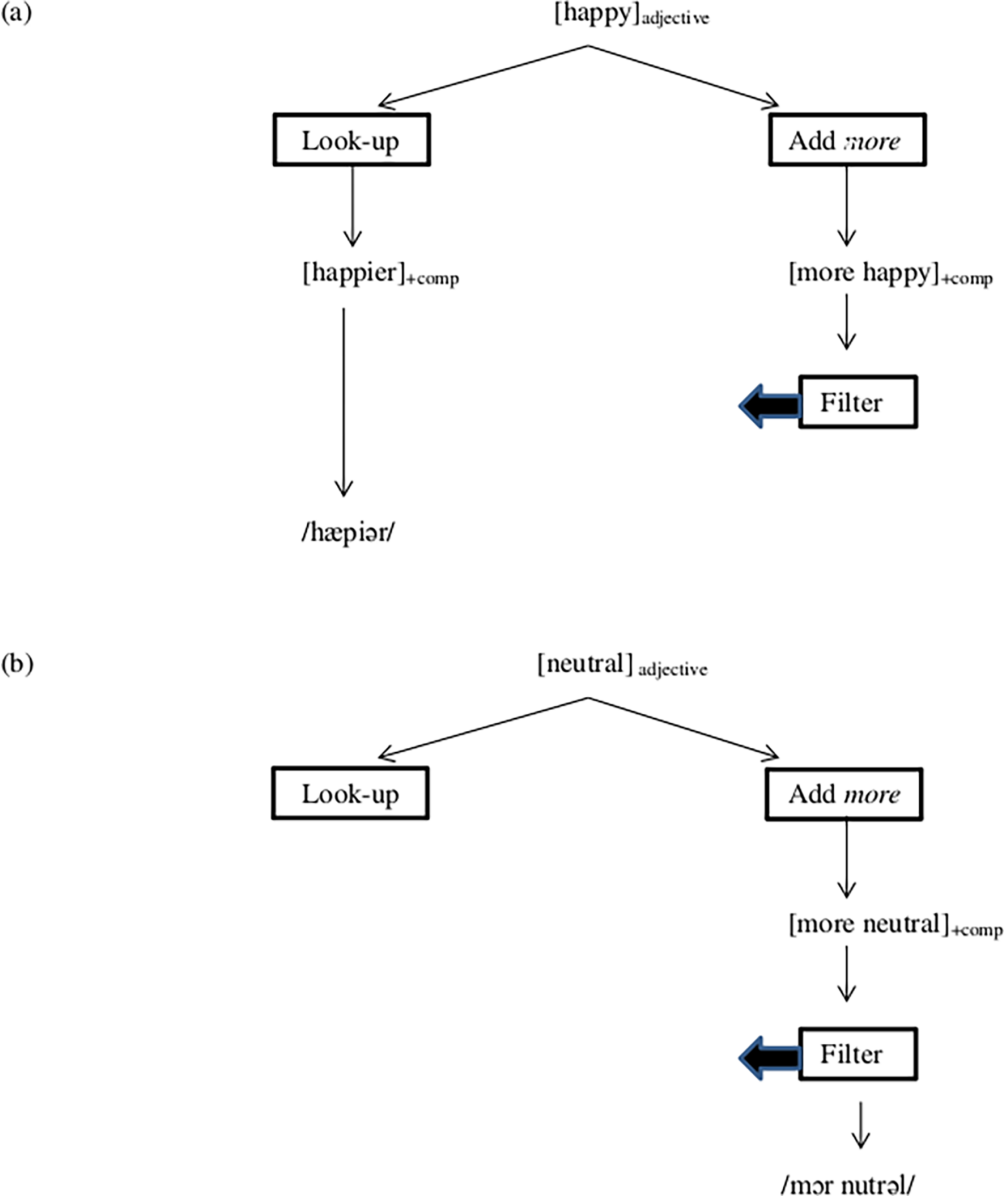
A schematic representation of the production of comparative adjectives.

While ERPs provide a high temporal resolution, leading to enhanced understanding of when morphological processing takes place, the *spatial information* they provide is rather limited (with the exception of high density ERP recordings and topographic analyses). Nevertheless, some studies have tried to identify the neural generators of the ERP effects of interest here (see [50] and [51] for discussion). As regards the fronto-centrally distributed N2 component that has been linked to conflict detection/conflict resolution mechanisms, Van Veen and Carter (2002) [52]—reviewing ERP and functional imaging studies—postulate that it is generated in the anterior cingulate cortex (ACC). Similarly, Lamm et al.’s (2006) [53] source-modelled neural generators of the N2 component in young adolescents as a function of cognitive control and linked it again to the ACC as well as to the orbito-frontal cortex. Furthermore, a recent single-case study [54] tested a patient with a damaged frontal aslant tract (FAT) which is formed by the white-matter fiber bundles descending from the anterior cingulate cortex and the pre-supplementary motor area to Broca’s area. During awake brain surgery, the patient was asked to form morphologically complex words, which elicited many morphological overapplications to forms that normally require lexically stored forms. The authors attributed this performance to the damaged FAT, which is supposed to lead to a deficit in the mechanisms that control the selection of lexicalized forms and rule-based processes during language production. These studies are part of a growing body of research suggesting that frontal brain areas are linked to cognitive control and error monitoring. The distribution of the negativity observed in the present study (as well as in our earlier ones) fits well with the distribution of previously reported frontal negativities linked to competition/control mechanisms suggesting that the particular case of control mechanism focused on here (see Fig 2) may also be mediated by a frontally distributed brain network.

As regards its *functional significance*, previous studies interpreted the enhanced negativity for the production of complex words as an index of morphological concatenation, viz. the composition of a stem plus an affix [21]. This interpretation straightforwardly accounts for the pattern of results obtained for the English past tense, for which regular forms (that involve affixation) elicited an enhanced negativity as opposed to irregular forms (that are not affixed). Applied to the current study, this account predicts a negativity for–*er* forms relative to analytic (*more*) comparatives, as the former (but not the latter) involve affixation. Our results, however, revealed the opposite pattern, namely a negativity for the production of analytic comparatives. Hence, our findings are not compatible with an interpretation of the negativity in terms of affix composition. An alternative functional interpretation builds on the fact that the production of comparative adjectives involves morpho-lexical competition between analytic and synthetic forms of expression. We specifically propose that the negativity reflects a constraint on grammatically-based computational processes, in this case *more* comparative formation. This is illustrated in Fig 2.

As illustrated in Fig 2A, the output of lexical look-up is unconstrained, which means that once an entry is found (for a synthetic comparative form), it is submitted to the speech output system and articulated, for example, as /hæpiər/ for the adjective *happy*. By contrast, accurate production of an analytic comparative form (illustrated in Fig 2B) requires an additional control process to check whether for a given a candidate form (e.g. *more neutral*) there is no equivalent stored form that would block the *more* form. We suggest that the elicited negativity we have observed in our ERP results signals this additional control/filter process involved in the production of analytic comparative forms.

We believe that other potential causes for this ERP effect are less likely. Conceptual and/or semantic encoding processes can be ruled out as the source for the enhanced negativity for analytic comparatives (relative to synthetic ones), because the two comparatives do not differ conceptually or in their meanings. Also, different adjective lemmas were involved when ERP responses to analytic versus synthetic forms were compared with each other (e.g., *happy* vs. *neutral*). However, the adverb control condition in which the same lemmas had to be produced as -*ly* adverb forms (e.g., *happily* vs. *neutrally*) did not elicit a negativity for MORE-adjective lemmas (relative to ER-adjective lemmas) indicating that the enhanced negativity for analytic comparatives cannot be attributed to any lexical differences of the lemmas involved. Furthermore, *more* and–*er* comparative responses differ in length, with the former (but not the latter) requiring the production of a two-word expression (*more neutral* vs. *happier*). We do not think that this contrast is responsible for the ERP differences obtained. If it were, the negativity should have disappeared when we compared *more* comparatives and negation of the same lemmas, because both conditions require two-word expressions (e.g., *more neutral* vs. *not neutral*). Our results (see bottom panel of Fig 1) show that this was not the case. Instead, the enhanced negativity for analytic comparatives was maintained relative to a control condition with stimuli of the same length (viz. negation) the production of which does not require any kind of output control for different means of expression. Given these findings, we maintain that the negativity for analytic comparative forms is an index of morpho-lexical competition.

We note that the account proposed here for the negativity obtained for analytic comparatives also applies to the results of production studies that tested other phenomena of morphological encoding. Recall that Budd et al. (2013) [21] and Festman and Clahsen (2016) [22] obtained this enhanced negativity for the production of regular past tense forms, Jessen et al. (2016) [24] for regular participles in German, and Budd et al. (2015) [23] for regular plurals inside compounds in English. What is common to the phenomena that elicited this negativity is that they involve grammatically-based computational processes that have to be prevented from over-application. These computational processes are not only the familiar cases of affixation but also include periphrastic forms such as *more* comparatives. These processes compete with stored lexical forms which may be unanalyzed wholes, for example suppletive forms, or may have internal structure, as for example irregular -*n* participles of German or–*er* comparatives in English. In any case, the proposed functional interpretation of the morphological encoding negativity in terms of competition is not dependent upon the notion of affix (de)composition or morpheme combination during word production, but would also be compatible with amorphous or supra-lexical models of morphological processing, e.g. [55] for which both recognition and production crucially involve competition between exponents.

## Conclusion

We found that the production of analytic comparative adjective forms (e.g. *more neutral*) elicited an enhanced negativity relative to the production of synthetic comparatives (e.g. *happier*). The onset latency of this brain response (~300ms) is consistent with previous findings from production studies [47–48] which have identified processes of morphological encoding to occur from this latency onwards. Control conditions ruled out non-morphological properties—in particular lexical and length differences between analytic and synthetic comparatives–as potential sources for this negativity. In previous research using the same silent-production-plus-delayed-vocalization paradigm that was used for the current study, this morphological encoding negativity has also been found for the production of regularly (relative to irregularly) inflected verbs and nouns in English and German. We propose a unified interpretation for the negativity obtained in these and the current study arguing that it reflects an output control mechanism during language production that prevents grammatically-based computational processes from over-applying.

Finally, we note that the ERP literature contains several reports of fronto-central negativities occurring in similar time-windows (~300-800ms) as the morphological encoding negativity, which have been interpreted as signaling conflict monitoring, cognitive control, and similar processes [50–51]. Consequently, we may speculate that the morphological encoding negativity is part of a family of negativities that reflect cognitive control processes more generally.

## Supporting information

S1 File ER-adjectives. (PDF)

S2 File MORE-adjectives. (PDF)

S1 Dataset Mean ERP amplitudes. (ZIP)

## References

[1] Janssen DP, Roelofs A, Levelt WJ. Inflectional frames in language production. Lang Cogn Process. 2002; 17(3): 209–236.

[2] Aronoff, M. Competition and the lexicon. In: Elia, A, Iacobini, C, Voghera, M, editors. Livelli di Analisi e fenomeni di interfaccia. Atti del XLVII congresso internazionale della società di linguistica Italiana. Roma: Bulzoni Editore; 2016. pp. 39–52.

[3] Pinker S. Words and rules: the ingredients of language 1st ed. New York, NY: Basic Books; 1999.

[4] Spalek K, Markus F. D, Bölte J. Is lexical selection in spoken word production competitive? Introduction to the special issue on lexical competition in language production. Language and Cognitive Processes. 2013; 28(5): 597–614.

[5] Bürki A. Sadat J., Dubarry A.-S., Alario X. Sequential processing during noun phrase production. Cognition. 2016; 146: 90–99. 26407338 10.1016/j.cognition.2015.09.002

[6] Hirschfeld G. Jansma B., Bölte J., Zwitserlood P. Interference and facilitation in overt speech production investigated with event-related potentials. Neuroreport. 2008; 19(12): 1227–1230. 10.1097/WNR.0b013e328309ecd1 18628670

[7] Misra M, Guo T, Bobb SC, Kroll JF. When bilinguals choose a single word to speak: Electrophysiological evidence for inhibition of the native language. J Mem Lang. 2012; 67(1): 224–237. 10.1016/j.jml.2012.05.001 PMC3820915 24222718

[8] Guo T, Liu H, Misra H, Kroll JF. Local and global inhibition in bilingual word production: fMRI evidence from Chinese-English bilinguals. Neuroimage. 2011; 56(4): 2300–2309. 10.1016/j.neuroimage.2011.03.049 21440072 PMC3741343

[9] Abutalebi J, Green DW. Neuroimaging of language control in bilinguals: neural adaptation and reserve. Biling. 2016; 19(4): 689–698.

[10] Runnqvist E, Strijkers K, Sadat J, Costa A. On the temporal and functional origin of L2 disadvantages in speech production: a critical review. Front Psychol. 2011; 2: 379 10.3389/fpsyg.2011.00379 22203812 PMC3241344

[11] Christoffels IK, Firk C, Schiller NO. Bilingual language control: an event-related brain potential study. Brain Res. 2007; 1147: 192–208. 10.1016/j.brainres.2007.01.137 17391649

[12] Strijkers K, Costa A, Thierry G. Tracking lexical access in speech production: electrophysiological correlates of word frequency and cognate effects. Cereb Cortex. 2010; 20(4): 912–928. 10.1093/cercor/bhp153 19679542

[13] Verhoef K, Roelofs A, Chwilla DJ. Role of inhibition in language switching: Evidence from event-related brain potentials in overt picture naming. Cognition. 2009; 110(1): 84–99. 10.1016/j.cognition.2008.10.013 19084830

[14] Ganushchak LY, Schiller NO. Brain error-monitoring activity is affected by semantic relatedness: An event-related brain potentials study. J Cogn Neurosci. 2008; 20(5): 927–940. 10.1162/jocn.2008.20514 18201131

[15] West R, Alain C. Event-related neural activity associated with the Stroop task. Brain Res Cogn Brain Res. 1999; 8(2): 157–164. 10407204 10.1016/s0926-6410(99)00017-8

[16] Müller O, Hagoort P. Access to lexical information in language comprehension: Semantics before syntax. J Cogn Neurosci. 2006; 18(1): 84–96. 10.1162/089892906775249997 16417685

[17] Rodriguez-Fornells A, van der Lugt A, Rotte M, Britti B, Heinze HJ, Münte TF. Second language interferes with word production in fluent bilinguals: brain potential and functional imaging evidence. J Cogn Neurosci. 2005; 17(3): 422–433. 10.1162/0898929053279559 15814002

[18] Schiller NO. Lexical stress encoding in single word production estimated by event-related brain potentials. Brain Res. 2006; 1112(1): 201–212. 10.1016/j.brainres.2006.07.027 16893534

[19] Schmitt BM, Münte TF, Kutas M. Electrophysiological estimates of the time course of semantic and phonological encoding during implicit picture naming. Psychophysiology. 2000; 37: 473–484. 10934906

[20] Elston-Güttler KE, Paulmann S, Kotz SA. Who's in control? Proficiency and L1 influence on L2 processing. J Cogn Neurosci. 2005; 17(10): 1593–1610. 10.1162/089892905774597245 16269099

[21] Budd MJ, Paulmann S, Barry C, Clahsen H. Brain potentials during language production in children and adults: An ERP study of the English past tense. Brain Lang. 2013; 127(3): 345–355. 10.1016/j.bandl.2012.12.010 23398779

[22] Festman J, Clahsen H. How Germans prepare for the English past tense: silent production of inflected words during EEG. Appl Psycholinguist. 2016; 37: 487–506.

[23] Budd MJ, Paulmann S, Barry C, Clahsen H. Producing morphologically complex words: An ERP study with children and adults. Dev Cogn Neurosci. 2015; 12: 51–60. 10.1016/j.dcn.2014.11.002 25541272 PMC6989782

[24] Jessen A, Fleischhauer E, Clahsen H. Morphological encoding in German children’s language production: Evidence from event-related brain potentials. J Child Lang. 2016; 44(2): 427–456. 10.1017/S0305000916000118 27018576

[25] Giraudo H., & Dal Maso S. The salience of complex words and their parts: Which comes first?. Frontiers in psychology. 2016; 7: 1778 10.3389/fpsyg.2016.01778 27917133 PMC5116555

[26] Pinker S, Ullman MT. The past and the future of the past tense. Trends Cogn Sci. 2002; 6(11): 456–463. 12457895 10.1016/s1364-6613(02)01990-3

[27] Emonds J. A transformational approach to English syntax: Root, structure-preserving, and local transformations New York: Academic Press; 1976.

[28] Barber C. Linguistic change in present-day English London et al.: Oliver & Boyd; 1964.

[29] Frank M. Modern English: A practical reference guide Englewood Cliffs, NJ: Prentice-Hall; 1972.

[30] Hilpert M. The English comparative language structure and language use. English Language and Linguistics. 2008; 12(3): 395–417.

[31] LaFave N. The most apt experimental investigation of English comparative and superlative formation. University of Pennsylvania Working Papers in Linguistics. 2015; 21(1): 1–12.

[32] Aronoff, M, Lindsay, M. Partial organization in languages: la langue est un système où la plupart se tient. Proceedings of the 8th Décembrettes; 2015. pp. 1–14.

[33] Poser WJ. Blocking of phrasal constructions by lexical items In: Sag IA, Szabolcsi A, editors. Lexical Matter. Stanford: CSLI; 1992 pp. 111–130.

[34] Graziano-King J, Cairns HS. Acquisition of English comparative adjectives. J Child Lang. 2005; 32(2): 345–373. 16045254 10.1017/s0305000904006828

[35] Dunbar E, Wellwood A. Addressing the “two interface” problem: Comparatives and superlatives. Glossa. 2016; 1(1:5): 1–29.

[36] Bölte J., Schulz C., Dobel C. Processing of existing, synonymous, and anomalous German derived adjectives: An MEG study. Neuroscience letters. 2010; 469(1): 107–111. 10.1016/j.neulet.2009.11.054 19944741

[37] Leminen A., Clahsen H. Brain potentials to inflected adjectives: Beyond storage and decomposition. Brain Research. 2014; 1543: 223–234. 10.1016/j.brainres.2013.10.038 24161829

[38] Bosch S., Krause H., Leminen A. The time-course of morphosyntactic and semantic priming in late bilinguals: A study of German adjectives. Bilingualism: Language and Cognition. 2017; 20(3): 435–456.

[39] Boyd J.K. Comparatively speaking: A psycholinguistic study of optionality in grammar University of California, San Diego, 2007.

[40] Kunter G. Effects of processing complexity in perception and production. The case of English comparative alternation. NetWordS. 2015: 32–36.

[41] Graziano-King J. English comparatives: a preliminary investigation Kingsborough Community College, CUNY, 2003.

[42] Clahsen H., Temple C. M. Words and rules in children with Williams syndrome In: Levy Y. & Schaeffer J. (Eds.), Language competence across populations. Erlbaum Press: Hillsdale, NJ, pp. 323–352.

[43] Gathercole V. More and more and more about more. Journal of Experimental Child Psychology, 1985; 40(1), 73–104. 4031787 10.1016/0022-0965(85)90066-9

[44] Baayen H, Piepenbrock R, van Rijn H. The CELEX lexical database (CD-ROM) Philadelphia, PE: Linguistic Data Consortium; 1993.

[45] Delorme A, Makeig S. EEGLAB: An open source toolbox for analysis of single-trial EEG dynamics. J Neurosci Methods. 2004; 134(1): 9–21. 10.1016/j.jneumeth.2003.10.009 15102499

[46] Koester D, Schiller NO. Morphological priming in overt language production: Electrophysiological evidence from Dutch. Neuroimage. 2008; 42: 1622–1630. 10.1016/j.neuroimage.2008.06.043 18674626

[47] Indefrey P, Levelt WJ. The spatial and temporal signatures of word production components. Cognition. 2004; 92(1–2): 101–144. 10.1016/j.cognition.2002.06.001 15037128

[48] Sahin NT, Pinker S, Cash SS, Schomer D, Halgren E. Sequential processing of lexical, grammatical, and phonological information within Broca’s area. Science. 2009; 326(5951): 445–449. 10.1126/science.1174481 19833971 PMC4030760

[49] Lavric A, Pizzagalli D, Forstmeier S, Rippon G. A double-dissociation of English past-tense production revealed by event-related potentials and low-resolution electromagnetic tomography (LORETA). Clin Neurophysiol. 2001; 112: 1833–1849. 11595142 10.1016/s1388-2457(01)00615-0

[50] Patel SH, Azzam PN. Characterization of N200 and P300: selected studies of the event-related potential. Int J Med Sci. 2005; 2(4), 147–154. 16239953 10.7150/ijms.2.147 PMC1252727

[51] Folstein JR, van Petten C. Influence of cognitive control and mismatch on the N2 component of the ERP: a review. Psychophysiology. 2008; 45(1): 152–170. 10.1111/j.1469-8986.2007.00602.x 17850238 PMC2365910

[52] van Veen V, Carter CS. The anterior cingulate as a conflict monitor: fMRI and ERP studies. Physiol Behav. 2002; 77(4–5): 477–482. 12526986 10.1016/s0031-9384(02)00930-7

[53] Lamm C, Zelazo PD, Lewis M. Neural correlates of cognitive control in childhood and adolescence: Disentangling the contributions of age and executive function. Neuropsychologia. 2006; 44: 2139–2148. 10.1016/j.neuropsychologia.2005.10.013 16310813

[54] Sierpowska J, Gabarrós A, Fernandez-Coello A, Camins À, Castañer S, Juncadella M, et al, A. Morphological derivation overflow as a result of disruption of the left frontal aslant white matter tract. Brain Lang. 2015; 142: 54–64. 10.1016/j.bandl.2015.01.005 25658634

[55] Giraudo H, Grainger J. Effects of prime word frequency and cumulative root frequency in masked morphological priming. Language and Cognitive Processes. 2000;15:421–44.

