# Supplementary material for: Morphological encoding beyond slots and fillers: An ERP study of comparative formation in English
Source: PLoS One. 2018 Jul 25;13(7):e0199897. doi: 10.1371/journal.pone.0199897 (PMC6059382; doi:10.1371/journal.pone.0199897)
Supplement: S1 File — (PDF) [file pone.0199897.s001.pdf]

S1. ER-adjectives

tasty, hungry, noisy, mild, cool, near, slim, tidy, lazy, loud, brave, empty, light, wise, dirty, angry, fresh, deep, narrow, soft, sweet, weak, clean, dry, dark, thin, wet, busy, thick, bright, kind, slow, quiet, rich, lucky, pretty, heavy, cold, late, funny, strong, happy, easy, simple, large
